# Astrocytic Kir4.1 channels and gap junctions account for spontaneous epileptic seizure

**DOI:** 10.1371/journal.pcbi.1005877

**Published:** 2018-03-28

**Authors:** Mengmeng Du, Jiajia Li, Liang Chen, Yuguo Yu, Ying Wu

**Affiliations:** 1 State Key Laboratory for Strength and Vibration of Mechanical Structures, School of Aerospace, Xi’an Jiaotong University, Xi’an, China; 2 State Key Laboratory of Medical Neurobiology, School of Life Science and Human Phenome Institute, Institutes of Brain Science, Institute of Science and Technology for Brain-Inspired Intelligence, Fudan University, Shanghai, China; 3 Department of Neurosurgery, Huashan Hospital, Shanghai Medical College, Fudan University, Shanghai, China; 4 Key Laboratory for NeuroInformation of Ministry of Education, University of Electronic Science and Technology of China, Chengdu, China; SUNY Downstate MC, UNITED STATES

## Abstract

Experimental recordings in hippocampal slices indicate that astrocytic dysfunction may cause neuronal hyper-excitation or seizures. Considering that astrocytes play important roles in mediating local uptake and spatial buffering of K^+^ in the extracellular space of the cortical circuit, we constructed a novel model of an astrocyte-neuron network module consisting of a single compartment neuron and 4 surrounding connected astrocytes and including extracellular potassium dynamics. Next, we developed a new model function for the astrocyte gap junctions, connecting two astrocyte-neuron network modules. The function form and parameters of the gap junction were based on nonlinear regression fitting of a set of experimental data published in previous studies. Moreover, we have created numerical simulations using the above single astrocyte-neuron network module and the coupled astrocyte-neuron network modules. Our model validates previous experimental observations that both Kir4.1 channels and gap junctions play important roles in regulating the concentration of extracellular potassium. In addition, we also observe that changes in Kir4.1 channel conductance and gap junction strength induce spontaneous epileptic activity in the absence of external stimuli.

## Author summary

Astrocytes are critical regulators of normal physiological activity in the central nervous system, and one of their key functions is removing extracellular K^+^. In recent years, numerous biological studies have shown that astrocytic Kir4.1 channels and gap junctions between astrocytes act as major K^+^ clearance mechanisms. Dysfunction of either of these regulatory mechanisms may cause generation of K^+^-induced seizures. However, it is unclear how and to what extent these two K^+^-regulating processes lead to spontaneous epileptic activity. These questions were addressed in the present study by constructing novel single astrocyte-neuron network models and a coupled astrocyte-neuron module network connected by an astrocyte gap junction based on existing experimental observations and previous theoretical reports. Simulation results first verified that either down-regulation of astrocytic Kir4.1 channels or a decrease of the gap junction strength between astrocytes causes neuropathological hyper-excitability and spontaneous epileptic activity. These results imply that dysfunctional astrocytes should be considered as targets for therapeutic strategies in epilepsy.

## Introduction

Temporal lobe epilepsy, which has a specific clinical presentation of seizures arising from the hippocampus [1], is a serious health risk to affected individuals. Extensive experimental and theoretical studies have shown that elevated K^+^ concentration in the extracellular microenvironment ([*K*^+^]_o_) may be linked to spontaneous epileptic seizure activity in the absence of external stimuli [2–6]. Astrocyte-mediated K^+^ buffering generally serves to maintain [*K*^+^]_o_, supporting normal neuronal electrical activities [7–10]. Mechanisms for K^+^ removal, regulated by astrocytes, can be broadly categorized as K^+^ uptake and K^+^ spatial buffering [11,12]. In the case of K^+^ uptake, excess K^+^ is absorbed primarily by astrocytes through Na^+^/K^+^-ATPase pumps and inwardly rectifying Kir4.1 channels or through Na^+^-K^+^-Cl cotransporters. K^+^ is spatially buffered via gap junctions, which redistribute excess K^+^ taken up from astrocytes in areas of excessive neuronal activity to astrocytes with lower K^+^ concentrations within the astrocytic network. Experimental data suggest that Kir4.1 channels play a prominent role in local uptake of extracellular K^+^ [8,13,14], and many experimental studies have observed that dysfunction of local uptake by astrocytes or inactivation of astrocytic gap junction protein expression cause the generation or spread of seizure activity [15–20]. It was recently reported that down-regulation or dysfunction of K^+^ uptake channels (Kir4.1) in astrocytes induces tonic-clonic seizures [7]. For example, low-intensity Schaffer collateral stimulation generates epileptiform activity in gap junction-deficient mice. Another experiment found that Tsc1 (TSC: Tuberous Sclerosis Complex) inactivation in astrocytes caused defects in astrocytic gap junction coupling and potassium clearance, leading to epilepsy in Tsc1GFAPCKO mice [21]. Furthermore, expression of the astrocytic gap junction proteins connexin 43 (Cx43) and connexin 30 (Cx30) is altered in epilepsy, and changes in gap junction communication have been observed in sclerotic hippocampal tissue of epileptic animal models [22]. Therefore, we investigated the dynamic mechanisms by which dysfunction of K^+^ uptake or gap junctions leads to changes in extracellular K^+^ concentration and resultant pathological epileptic discharges using a theoretical study with a computational astrocytic-neural network model.

Recent studies have applied theoretical analysis approaches to explore the relationship between astrocytic modulation of [*K*^+^]_o_ and the initiation or maintenance of epileptic seizure activity [23–32]. Cressman J. R. et al. provided a simplified astrocytic K^+^ uptake function that incorporates both passive and active local K^+^ uptake into a single sigmoidal response function that depends solely on extracellular K^+^ concentration [23–27]. Hübel studied the mechanism of extracellular K^+^ uptake in astrocytes using a phenomenological equation for the rate of K^+^ uptake, which depends entirely on extracellular K^+^ concentration [30]. Øyehaug L et al. modeled the mechanism of K+ uptake from the perspective of volume dynamics [28,29], modeling the cotransporters (Na^+^/K^+^/2Cl and K^+^/Cl^-^ cotransporters) and the Na^+^/K^+^-ATPase pump of astrocytes. However, these model systems did not adequately reflect the complexity of astrocytes. For example, in addition to cotransporters and the Na^+^/K^+^-ATPase pump, the inwardly rectifying K^+^ channels (Kir4.1) in astrocyte membranes and inter-astrocyte spatial buffering also contribute to extracellular K^+^ removal. Sibille J et al. developed mathematical models of Kir4.1 channels that depend on both the intra-astrocyte K^+^ concentration and the astrocytic membrane potential [31,32]. In addition, they verified the prominent role of Kir4.1 channels in regulating [K^+^]_o_ during repetitive stimulation using their mathematical model.

It is worth mentioning that none of the aforementioned studies considered the relationship between Kir4.1 channel conductance and the occurrence of spontaneous epileptic seizures. Morphologically, previous studies of the extracellular K^+^ local buffer mechanisms in astrocyte-neuron networks have shown that astrocytes have a 1:1 quantitative relationship with neurons; however, experimental data show that one cortical neuron is typically surrounded by multiple astrocytes [20,33,34]. In support of this, in 2009, Azevedo et al. reported that the ratio of neurons to glia is approximately 1:4 in the human cerebrum [33].

In light of these findings, we constructed a model of an astrocyte-neuron network consisting of a single compartment neuron connected to four surrounding astrocytes with Kir4.1 channels and Na^+^/K^+^-ATPase pumps. Extracellular potassium ions diffused in and out of the space between the neuron and astrocytes. In addition, we developed a novel configuration for astrocytic gap junctions, connecting two astrocyte-neuron network modules. The model function parameters of the gap junction are based on a nonlinear regression fit of a set of experimental data previously published [17]. The model simulation results were validated by comparison with published experimental data [17]. We conducted a series of simulations using the single astrocyte-neuron network models and the coupled astrocyte-neuron module network and showed that changes in Kir4.1 channel conductance and gap junction strength induce spontaneous epileptic activity in the absence of external stimuli.

## Results

### Astrocytic K^+^ uptake by Kir4.1 channels

**Fig 1:**
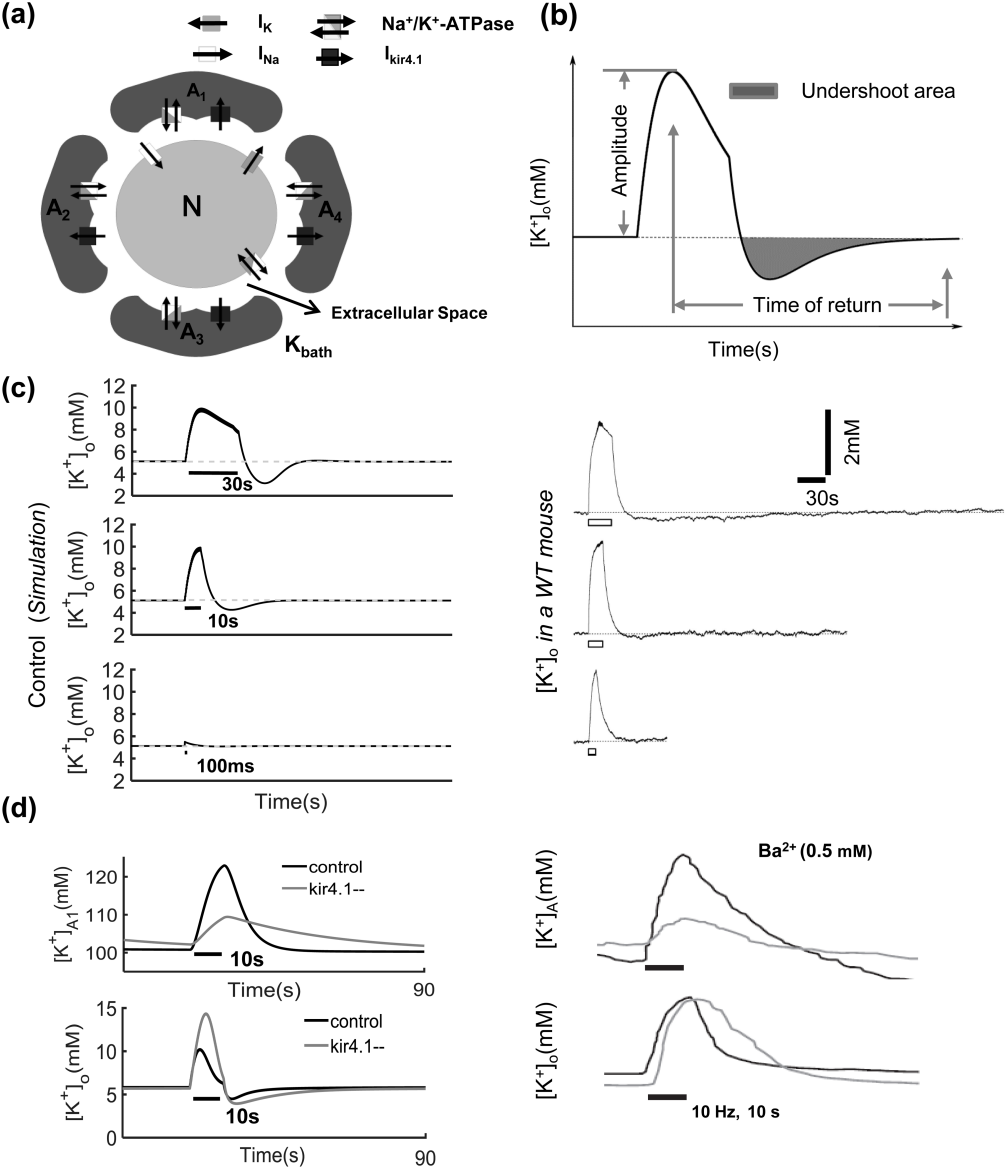
**(a)** A schematic of the single neuronal model dynamics. **(b)** The concept of undershoot is proposed here, with the total surface area of the undershoot estimated (gray area). The duration of the undershoot (“time of return”) corresponds to the time needed for [*K*^+^]_o_ to recover to the basal level after the end of the stimulation. **(c)** The time series of extracellular K^+^ concentration after different sine stimulation periods when Kir4.1 channels are in controlled states, such as 30 s, 10 s, 100 ms, from simulation results (left) and experiments interpolated for comparison (right) from Fig 3 in Chever O et al [35]. **(d)** K^+^ concentration in the extracellular space ([*K*^+^]_o_) and astrocyte ([*K*^+^]_A_) response to Kir4.1 channel in controlled and blocked states during and after 10 s sine stimulus spike. Simulation results (1); experiments correspond to results interpolated from Fig 7 in Ballanyi K et al [36]. For other parameter values, see Table 1. In addition, *ɛ* = 1.2/s^-1^ and *K*_*bath*_ = 4 mM.

To validate the role of Kir4.1 channels in regulating astrocytic and neuronal extracellular K^+^ concentration, a model was constructed representing astrocyte-neuron network modules consisting of a single compartment neuron connected to four surrounding astrocytes with Kir4.1 channels and Na^+^/K^+^-ATPase pumps (Fig 1(a)). Based on evidence from previous studies [23,25,26,30,31], there exist several key factors mediating K^+^ concentration in the extracellular space: K^+^ currents across the neuronal membrane, Na^+^/K^+^-ATPase pumps for neurons and astrocytes, Kir4.1 channels in astrocytes, spatial diffusion of K^+^ in the extracellular space, and others (illustrated in Fig 1(a)). During an action potential, K^+^ is released by neurons at the extracellular space, followed by K^+^ flowing into adjacent astrocytes via the Na^+^/K^+^-ATPase pump and Kir4.1 channels. It is important to note that the Kir4.1 current is larger in magnitude than the outward Na^+^ current from the Na^+^/K^+^-ATPase pump, resulting in overall depolarization of the astrocytic membrane (Fig 1(b)).

Effects of Kir4.1 channel blockage on extracellular K^+^ regulation were first tested by setting the Kir4.1 conductance to 45.0 *pS* for the normal (control) condition. Sine stimulus inputs (the stimulus amplitude and frequency are *1μA*/*cm*^2^ and 10 *Hz*, respectively; here, 10 Hz was used to verify our results in comparison with previous experimental recordings [35]) with different durations, e.g., 30 *s*, 10 *s* and 100 *ms*, were applied to the neuron when Kir4.1 channels were in the control state. Note here that a 100 *ms* stimulus resulted in a small undershoot and a very short recovery to the [*K*^+^]_o_ baseline. As the duration increased, both the undershoot area and recovery time increased. Compared with 100 *ms*, the 10 *s* and 30 *s* stimulations required significantly longer times to recover to the baseline, as shown in Fig 1(c). This result is consistent with an experimental trend observed *in vivo* in the mouse hippocampus [35].

We next tested our model with Kir4.1 channel inhibition (*g*_*kirA*_ = 0.01 *pS*). We observed that extracellular K^+^ concentration displayed a more pronounced undershoot after long-lasting sine stimulation (10 *Hz*, 10 s). The maximum amplitude of extracellular K^+^ concentration for *g*_*kirA*_ = 0.01 *pS* was higher than that for *g*_*kirA*_ = 45.0 *pS* during a 10 *s* external stimulation input (see lower panel in Fig 1(d)). During the 10 *s* post-stimulus recovery period, extracellular K^+^ initially dropped to below the baseline levels before returning gradually to the resting-state equilibrium concentration, taking even longer to recover to the K^+^ baseline concentration, as shown in the lower panel of Fig 1(d). Furthermore, it was observed that in response to stimulus under control conditions, astrocytes responded with a rapid increase in intra-astrocyte K^+^ concentration with an accompanying high amplitude, while the baseline K^+^ concentration was low. On the contrary, the K^+^ concentration in astrocytes rose much more slowly to a lower peak concentration when Kir4.1 was blocked, as shown in the top panel of Fig 1(d). These computational results were all in agreement with previously recorded experimental results [35,36].

**Fig 2:**
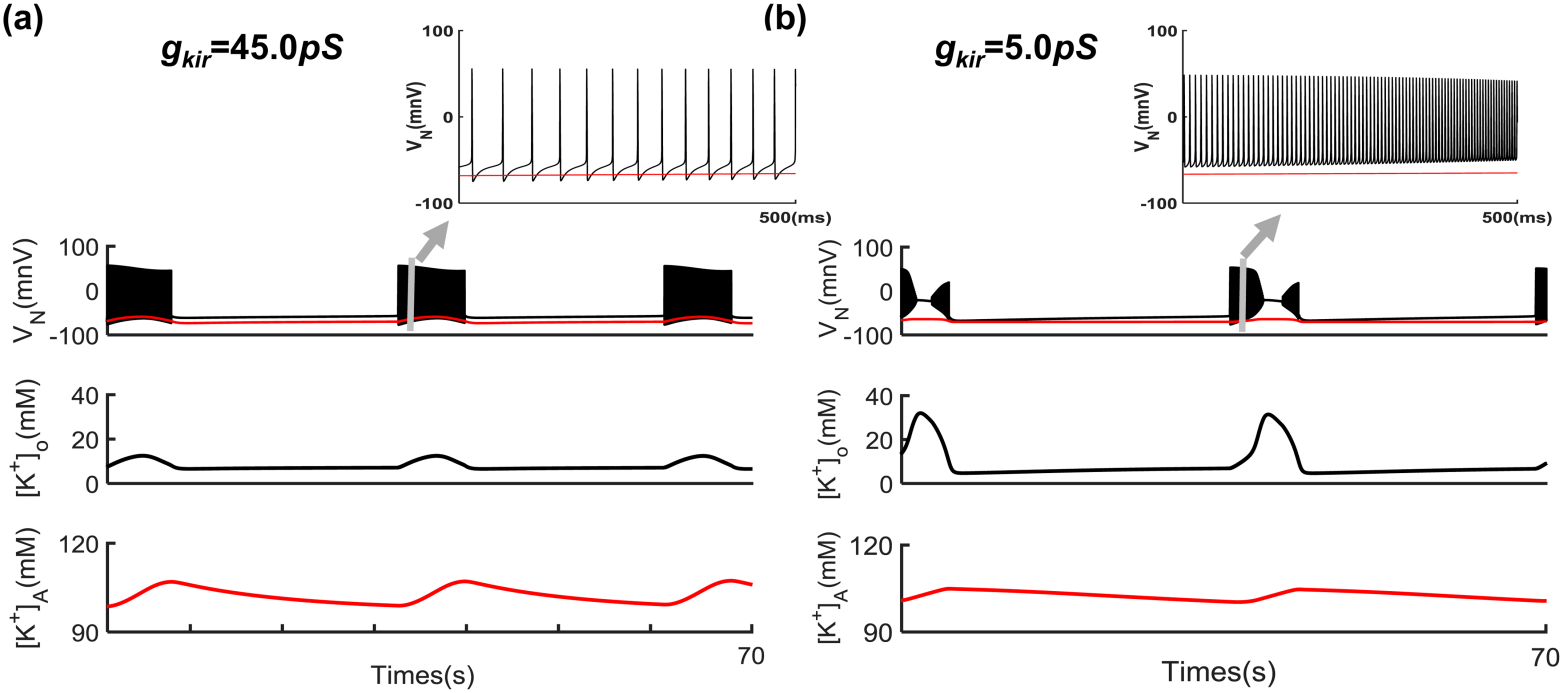
Decreased astrocytic Kir4.1 channel conductance *g*_*kir*_ induces spontaneous periodic epileptic seizures in the absence of external stimuli. Time trains of the neural and astrocytic membrane potential (*V*_*N*_ (mV)) and extracellular K^+^ concentration ([*K*^+^]_o_) (black lines); membrane potential *V*_*A*_ (mV) and K^+^ concentration ([*K*^+^]_A_) for astrocyte (red lines) when *g*_*kir*_ is 45.0 *pS* **(a)** and 5.0 *pS* **(b)**, found from the equations presented in the model section. *K*_*bath*_ = 8 mM, and other parameters used are the same as in Fig 1.

Additionally, the critical value of *g*_*kir*_ was examined for the generation of spontaneous epileptic seizures in the absence of external stimuli. Model simulations suggested that spontaneous periodic epileptic activity is induced when *g*_*kir*_< = 7.0 *pS* without external stimulation (Fig 2(b)), characterized by prolonged (2–15 s) interruptions in population spike generation. During these interruptions, neuronal firing is suppressed rather than desynchronized. Bikson et al. have observed this depolarization block in pyramidal cells during electrographic seizures in rat hippocampal slices (Fig 1d [3]). Fig 2 shows the time sequences of neuronal and astrocytic membrane potentials, time courses of K^+^ concentration in the extracellular space and intra-astrocytes for *g*_*kirA*_ = 45 *pS* and 5.0 *pS*. These results indicate that neurons fire normally only when the Kir4.1 channel conductance is adequate. A low Kir4.1 channel conductance (Fig 2(b)) results in spontaneous periodic seizures in the astrocyte-neuron network module. Fig 2 also shows that spontaneous periodic epileptic firing is accompanied by higher amplitude oscillations of extracellular K^+^ concentrations and lower peak intra-astrocyte K^+^ concentrations compared with normal neuronal discharges.

### Gap junction deficiency causes a delay in extracellular K^+^ buffering

**Fig 3:**
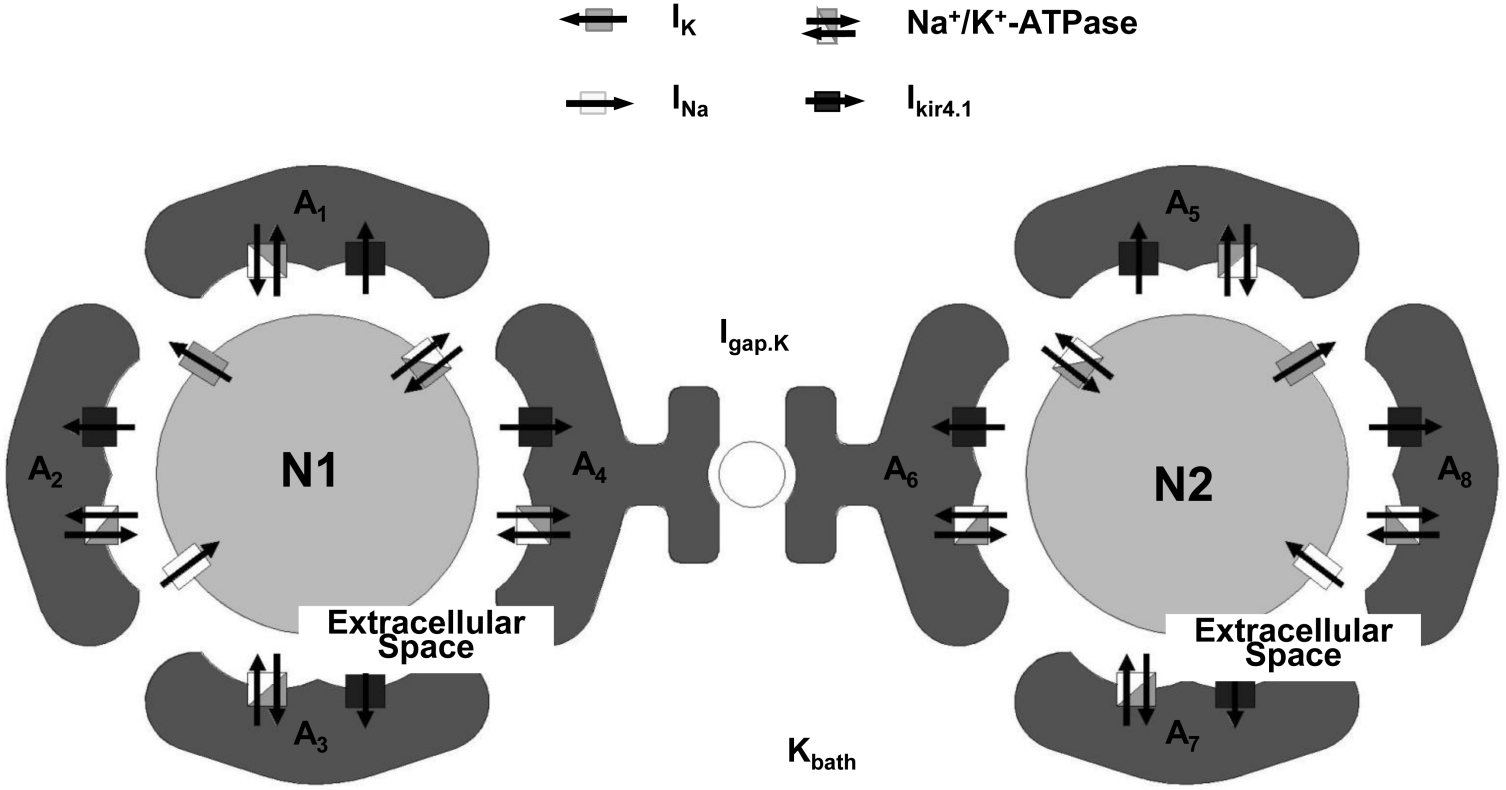
A conceptual diagram of the gap junction-mediated astrocytic-neural network model.

Fig 3 presents a new model in which astrocyte gap junctions connect two astrocyte-neuron network modules. This network model was constructed to examine the K^+^ spatial buffering mechanisms dominated by astrocytic gap junctions. In this model, individual neurons are surrounded by 4 astrocytes, and they share the same bath K^+^ concentration. During action potential generation, neurons release K^+^ into the extracellular space, and K^+^ enters adjacent astrocytes via the Na^+^/K^+^-ATPase pump and Kir4.1 channels. Subsequently, in astrocytes with higher concentrations, K^+^ may flow into adjacent astrocytes via inter-astrocytic gap junctions due to the electrochemical driving force.

**Fig 4:**
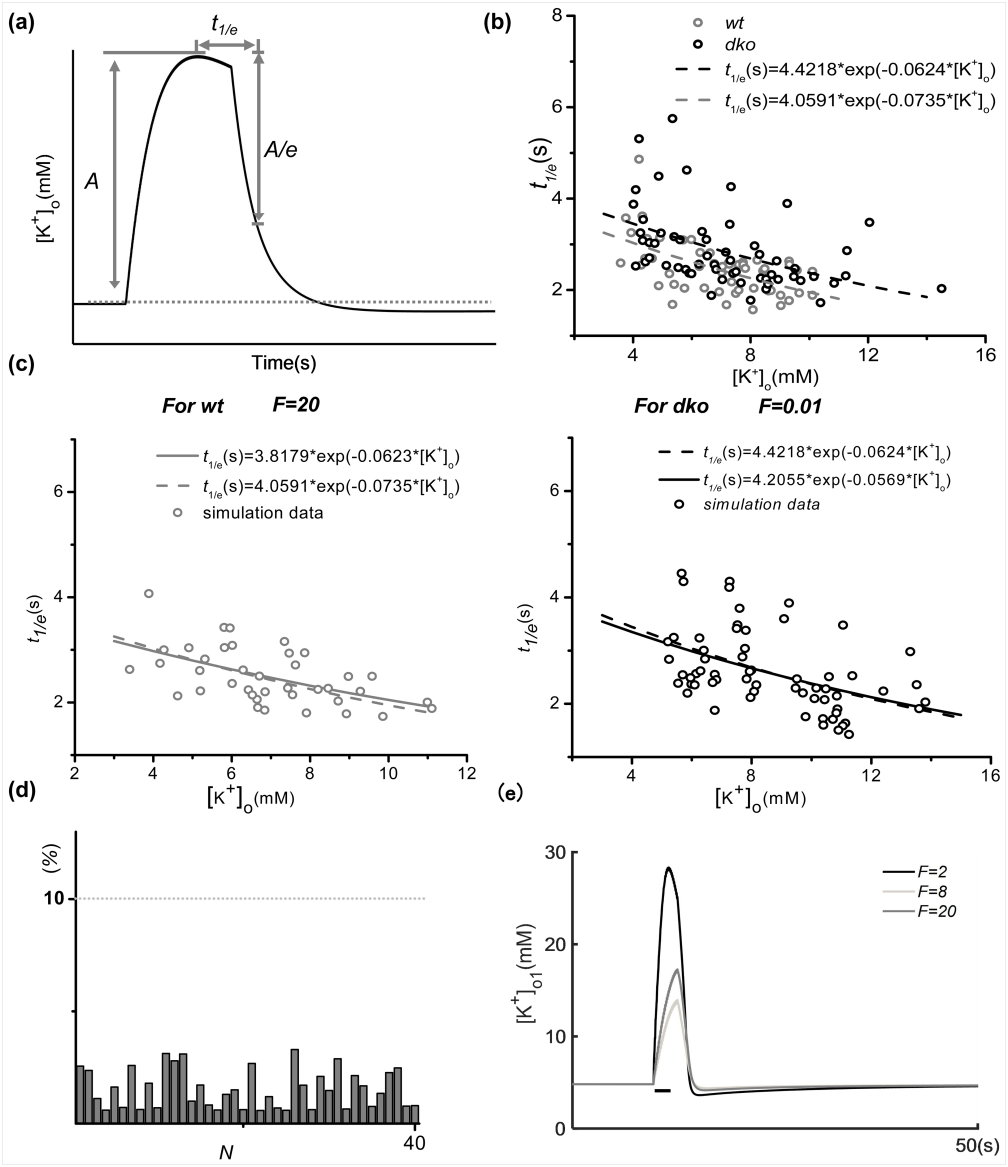
**(a)** The decay factor *t*_*1/e*_ is proposed here. **(b)** A schematic diagram of extracellular K^+^ concentration ([*K*^+^]_o_) and the decay factors *t*_*1/e*_ as published in experimental results (see Fig. 5B from [17]). Black spots and gray spots are experimental data obtained from connexin30^-/-^ rats and control gap junction rats, respectively. **(c)** The relationship between extracellular K^+^ concentration and decay factor *t*_*1/e*_ in control conditions or the gap junction-deficient state. The dashed lines and solid lines denote experimental fitted curves (see Fig. 5B from [17]) and fitted curves from numerical simulation data, respectively. **(d)** Statistical error for experimental and numerical fitted curves for different strengths of gap junctions. **(e)** K^+^ undershoot in the extracellular space with different gap junction strengths after the same external stimuli sequence. Other parameters used are the same as in Fig 1.

To validate whether this type of inter-astrocytic gap junction behaves the same way as those in experimental observations, the decay dynamics of extracellular K^+^ concentration ([*K*^+^]_o_) was examined in response to the sine stimulus trains (10 s, 20 Hz). We chose 20 Hz rather than the 10 Hz used above in order to compare the results in our model with previously published experimental results (Fig. 5B in [17]). The results demonstrated that after the stimulus trains, [*K*^+^]_o_ initially decayed very rapidly, followed by a prolonged phase of slower decay, often associated with an undershoot [37] (Fig 4(a)). To quantify the faster K^+^ decay phase, the time at which the [*K*^+^]_o_ amplitude has decayed to *1/e* of its initial maximal amplitude is defined as the decay time constant (*t*_*1/e*_), providing a descriptive parameter for the decay process. Anke Wallraff [17] observed an inverse correlation between *t*_*1/e*_ and [*K*^+^]_o_, which is well fitted by a power-law function (Fig 4(b)). Fig 4(b) illustrates the experimentally observed fitted curve functions *t*_*1/e*_ = 4.0591exp(-0.0735[*K*^+^]_o_) for normal gap junctions and *t*_*1/e*_ = 4.4218exp(-0.0624[*K*^+^]_o_) for deficient gap junctions obtained from rat hippocampal slice recordings. Clearly, deficient gap junctions are characterized by a larger decay time constant, suggesting slower removal of K^+^ load [17]. Here, the proposed model simulated this process by applying the sine stimulus trains (10 s, 20 Hz, the stimulus amplitude is randomly chosen from 1 to 60*μA*/*cm*^2^ to induce different spiking patterns and potassium diffusion dynamics) to neuron 1 (N1). The K^+^ concentration *K*_*bath*_, the spatial diffusion coefficient *ε* and the Na^+^/K^+^-ATP pump strength *ρ* were set to different values to study their effect on the correlations between the diffusion time constant t_1/e_ and extracellular K^+^ concentration, as shown in Fig 4c. The Kir4.1 channel conductance was set to 45.0 *pS* for Fig 1c (for values of the other parameters, see Table 1). By setting the strength of the gap junction to *F* = 20.0 and 0.01, the model simulation reproduced the wild-type control (Fig 4c) and *dko* deficient (Fig 4d) gap junctions for neuron 1 (N1). We then used the nonlinear least-squares method to fit the relation curve of *t*_*1/e*_ and [*K*^+^]_o_ that was obtained by the coupled astrocyte-neuron network modules. The fitted curve function for normal gap junctions is *t*_*1/e*_ = 3.8179exp(-0.0623[*K*^+^]_o_) (left panel, Fig 4(c)). The fitted curve function for deficient gap junctions is *t*_*1/e*_ = 4.2055exp(-0.0569[*K*^+^]_o_) (right panel, Fig 4(c)). The simulated fitted curve functions are quantitatively similar to experimental observations. In addition, to validate the physiological significance of the model constructed in this section and the reliability of our numerical results, statistical analysis was conducted on the error between the experimental and numerical fits for different gap junction strengths, as shown in Fig 4(d). Here, the fit error is the mean absolute error (calculations shown in S1 Text). The fit error between the numerical and experimental data is far less than 10%. Considering the experimental accuracy and other environmental factors, we believe that the gap junction-mediated coupled model presented herein is within the permissible error range. In addition, different strengths of gap junctions were applied between astrocytes, e.g., *F* = 20 and 0.01(Fig 4(e)). The results show that decreased gap junction strength induces higher [*K*^+^]_o_ peaks, and if the strength of gap junctions experiences a strong decrease, [*K*^+^]_o_ will have a larger undershoot and an increased baseline recovery time.

**Table 1:** Model parameters.

Parameter	Value and units	Description
*C*_*N*_	1*μF*/*cm*^2^	Membrane capacitance of neuron
*g*_*Na*_	100*mS*/*m*^2^	Conductance of the persistent sodium current
*g*_*K*_	40*mS*/*m*^2^	Conductance of the potassium current
*g*_*AHP*_	0.01*mS*/*m*^2^	Conductance of the after-hyperpolarization current
*g*_*KL*_	0.05*mS*/*m*^2^	Conductance of the potassium leak current
*g*_*NaL*_	0.05*mS*/*m*^2^	Conductance of the sodium leak current
*g*_*Cl*_	0.05*mS*/*m*^2^	Conductance of the chloride leak current
*g*_*Ca*_	0.1*mS*/*m*^2^	Calcium conductance
*V*_*Ca*_	120*mV*	Reversal potential for the chloride current
_*v*1_	7.0	The volume rate of extracellular space and neuron
*v*_2_	3.0	The volume rate of extracellular space and astrocyte
[*Cl*]_*i*_	6.0*mM*	Intracellular chloride concentration
[*Cl*]_*o*_	130.3*mM*	Extracellular chloride concentration
C_A_	15*pF*	Membrane capacitance of astrocyte
g_lA_	5.0*mM*^−1^*ms*^−1^	Astrocytic leak conductance

The above simulations applied the exponential diffusion function observed in experimental data reported by Anke Wallraff [17]. While this is a reasonable method to validate our overall model, it does not necessarily justify modeling gap junction diffusion as an exponential function of intracellular K^+^. Therefore, we performed a new set of numerical simulation experiments to address this concern. We chose several different types of diffusion functions for our model network. For example, a linear function (*J*_*i*−*j*_ = *F*([*K*^+^]_*Ai*_ − [*K*^+^]_*Aj*_) (the numerical simulation results and regression fitting curve of the network model are shown in S1B Fig, compared with our original model results S1A Fig) and a threshold-nonlinear function (*J*_*i*−*j*_ = *F* * (1 + tanh ((|[*K*^+^]_*Ai*_ − [*K*^+^]_*Aj*_|—*K*_*thr*_)/*K*_*Scale*_))) (see S1C Fig). The fitting error ranges of the three functions are shown in S1D Fig. The simulation results suggest that the dynamic properties of potassium ion channel diffusion faithfully follow the exponential decay function.

**Fig 5:**
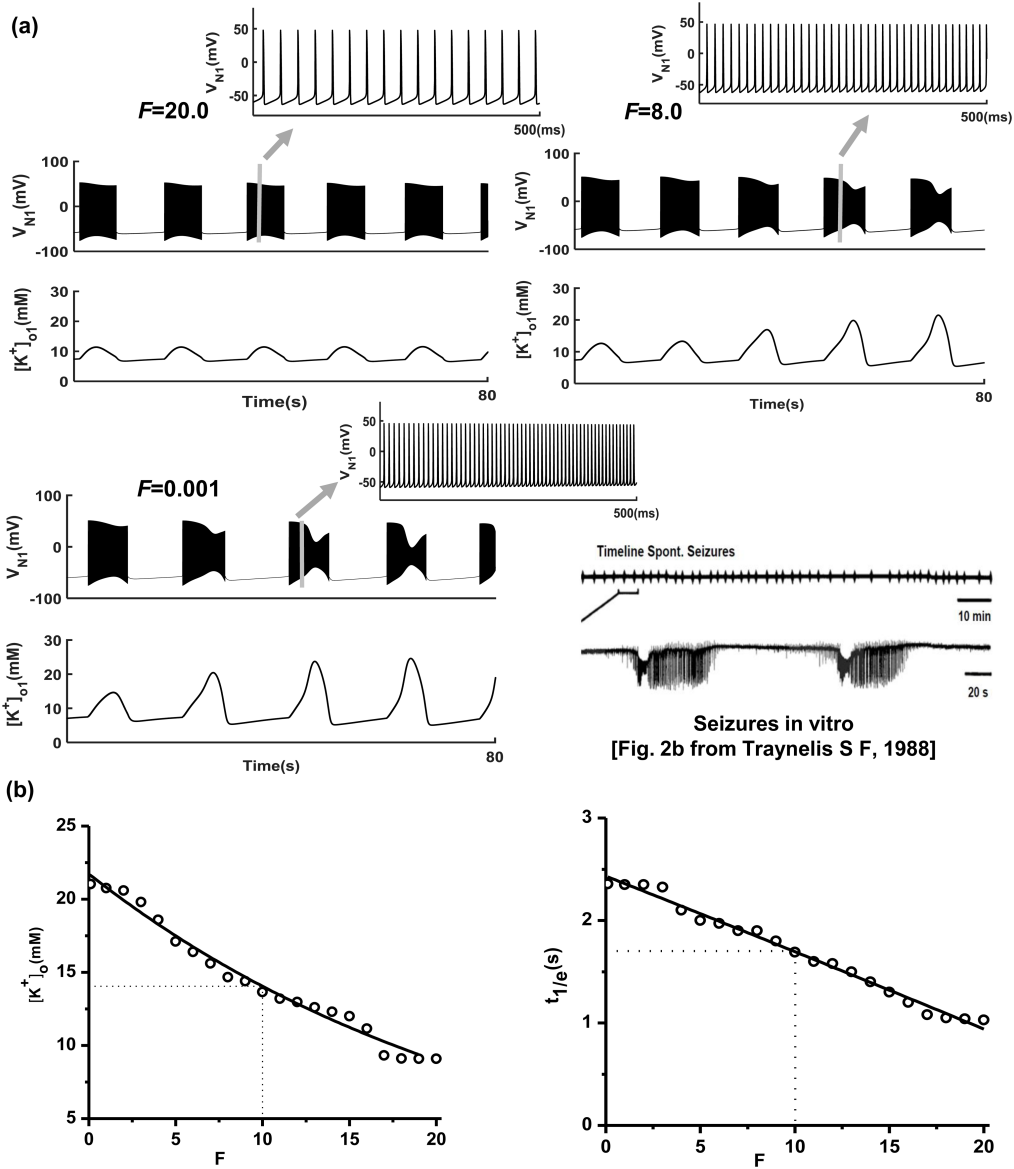
**(a)** Decreased gap junction strength *F* induces spontaneous periodic epileptic seizures in the absence of external stimuli. Normal discharge states of neurons when *F* is 20.0; periodic spontaneous seizures present at 50 *s* when *F* is 8.0 and at 24 *s* when *F* is 0.01. Experimental dual recordings of membrane potential at a 100 *μm* depth within hippocampal slices in the brain (data from [41,42]) **(b)** Schematic relationship of gap junction strength *F* and extracellular K^+^ concentration ([*K*^+^]_o_) or the decay factors *t*_*1/e*_. Other parameters used are the same as in Fig 2.

Numerous experiments have observed that the coupling-deficient astrocytic network (with depression of connexin43 expression or connexin30 expression) in the nervous system is related to generation of spontaneous seizures [17,22,38–40]. To verify the relationship between the dysfunction of the gap junctions and spontaneous epileptic discharges in the absence of external stimuli, we used the coupled two astrocyte-neuron network modules. Model simulations suggested that spontaneous epileptic activity is induced when *F*< = 10.0 *pS* without external stimulation (Fig 5(a)). Fig 5(a) shows the time sequences of the neuronal membrane potentials (*V*_N1_) and the K^+^ concentration in the extracellular space ([K^+^]_o1_) for *F* = 20.0, 8.0 and 0.001. The neuron (N1) exhibited normal spontaneous slow oscillation activity when the gap junction strength *F* was equal to 20.0. However, the activity changed into spontaneous epileptic seizures after certain time periods when the value of *F* decreased. For example, the neuron (N1) changed into spontaneous seizures firing at 50 s when *F* was 8.0. Specifically, neurons rapidly enter spontaneous epileptic discharge with smaller gap junction strengths. As such, the neuron (N1) exhibited spontaneous seizure firing at 50 s when *F* was 8.0, while entering spontaneous seizure firing at 24 s when *F* was 0.001 (Fig 5(a)). This type of spontaneous epileptic seizure has been observed in various experimental recordings [2,41,42]. Fig 5(b) summarizes the schema of astrocytic gap junction strength *F* and [*K*^+^]_o_, or *t*_*1/e*_ when there is no external stimuli to the neurons, the Kir4.1 channel conductance is 45.0 *pS* and other parameters are fixed, as in Table 1. [*K*^+^]_o_ and *t*_*1/e*_ for N1 gradually decrease as *F* increases. These relationships can be used to explain the extracellular K^+^ concentration requiring more time to recover to the baseline when the gap junction is deficient. The above results confirm that decreasing astrocytic gap junction strength induces spontaneous epileptic seizures in the astrocyte-neuron network in the absence of external stimulation. Furthermore, neurons will rapidly enter into spontaneous epileptic seizures with smaller gap junction strength.

## Discussion

Potassium clearance is widely accepted as a primary function of astrocytes. Several computational models have investigated the buffering mechanism for extracellular K^+^ accompanied by neural activities [23,25,26,28,30,31,43]. A model accounting for K^+^ concentration in the extracellular space and astrocytic compartments has been used to quantify involved astrocytic ion channels and transporters (Na^+^/K^+^-ATPase, NKCC1, etc.) [28,29]. While NKCCl has been recently shown to not be involved in activity-dependent K^+^ clearance in hippocampal slices [15], astrocytic Kir4.1 channels are crucial for the recovery of basal extracellular K^+^ levels and neuronal excitability during external stimulation [31]. To unravel how the acute role of astrocytes in [*K*^+^]_o_ homeostasis induces spontaneous epileptic discharge and maintains normal electrophysiological activity in the astrocyte-neuron system, we constructed an astrocyte-neuron network module consisting of a single compartment neuron connected to four surrounding astrocytes with Kir4.1 channels and Na^+^/K^+^-ATPase pumps. Extracellular potassium ions were allowed to diffuse in and out of the space between the neuron and astrocytes. In addition, we have built a new model for astrocytic gap junctions, enabling connection of two astrocyte-neuron network modules. The model function parameters were based on a nonlinear regression fit based on a set of experimental data published previously [17]. The model simulation results were validated by comparison with the published results [17]. We first verified that spontaneous periodic seizures can be induced directly in the absence of stimuli when the astrocytic Kir4.1 channel conductance or the strength of gap junction-mediated K^+^ spatial buffering decreases to a certain level.

We first simulated the specific dynamic characteristics of astrocytic Kir4.1 channel conductance inducing spontaneous epileptic activity in the absence of external stimulus input. To mathematically examine K^+^ dynamics in neurons, astrocytes and extracellular spaces, we constructed an astrocyte-neuron network module consisting of a single compartment neuron connected to 4 surrounding astrocytes. The results demonstrated that the K^+^ undershoot of the extracellular space increases after a period of stimulus in Kir4.1 knockouts [15,35,44]. We verified these results using the proposed computational model compared to controls and found that K^+^ concentration in the extracellular space exhibits an increased undershoot after a 10 s sine stimulus sequence, taking a longer time to return to the baseline in Kir4.1 channel-deficient conditions. Moreover, the baseline K^+^ concentration in astrocytes is higher and rises more slowly to a decreased peak value after an external stimulus train compared to controls. The high baseline K^+^ concentration in astrocytes is due to the normal function of the Na^+^/K^+^-ATPase pump. In particular, spontaneous epileptic activity was directly induced in the single astrocyte-neuron network module without external stimuli when the conductance of Kir4.1 channels was lower than a certain threshold, further demonstrating that down-regulation of astrocytic Kir4.1 channels is closely related to neuropathological hyper-excitability [8,45,46]. At the same time, our single astrocyte-neuron network correctly predicts that when there is a partial blockage of Kir4.1 channels in astrocytes, rapid instantaneous neuronal seizure activity is induced. Moreover, it is possible that astrocytic Kir4.1 channels modulate extracellular K^+^ as a means of regulating synaptic activity.

The dynamic characteristics of astrocytic gap junction strength pertaining to spontaneous epileptic activity in the absence of external stimuli were first simulated in this study. Gap junctions in astrocytes appear to play a dual role: on the one hand, they counteract the generation of hyperactivity by facilitating the decrease of extracellular K^+^ levels; on the other hand, they constitute a pathway for energetic substrate delivery that fuels neuronal hyper-excitability [16]. Thus far, research involving the effects of astrocytic networks coupled with gap junctions on extracellular K^+^ spatial buffering is still in the biological experimentation stage. Previous studies have examined the role of astrocytic gap junctions in potassium buffering with respect to epileptic seizures [17,22]. We presented a new exponential function model for astrocyte gap junctions that connect two astrocyte-neuron network modules. To verify the validity of the proposed model of gap junctions, we compared two groups of data of the fitted exponential function curves of *t*_1/e_ vs [*K*^*+*^]_o_ obtained from numerical simulation data based on the coupled astrocytic-neural network modules and previous experimental recordings [17]. Both control conditions and deficient gap junction cases were compared while sine stimulus trains were applied to neuron 1 (N1). We discovered that the curve fitting error of numerical and experimental data was within a reasonable range. In addition, the numerical results validated the finding that gap junction-mediated K^+^ spatial buffering is slower in the absence of astrocytic gap junctions compared to wild-type rats. At the same time, the neuron entered a spontaneous epileptic state more quickly at weaker gap junction strengths. Finally, we summarized the schema of the astrocytic gap junction strength *F* and [*K*^*+*^]_o_ or *t*_*1/e*_ when there is no external stimulation to either of the neurons, and this relationship can be used to explain the observation that extracellular K^+^ requires more time to return to the baseline when the gap junction is dysfunctional. Indeed, our results reproduced experimental observations wherein expression-deficient astrocytic gap junction proteins (Cx43 or Cx30) distorted neuronal information processing and the generation of spontaneous epileptiform events [16,47]. Our coupled astrocyte-neuron network modules predict that with partially blocked astrocytic gap junctions, neuronal seizure activity will gradually develop after a delay of tens of seconds. This finding could represent a protective mechanism to prevent K^+^ accumulation from reaching neurotoxic levels. These results can be used as a basis for performing further analysis of the characteristics of sodium, calcium, the power factor and other factors in the network, as well as the mutual influences among them.

In this study, we considered the gap junction between one of two adjacent astrocytes. *In vivo* many glial cells surrounding the firing neuron will buffer potassium into adjacent glial cells that have lower concentrations. We have chosen several other different diffusion functions (including an exponential function, a linear function and a threshold-nonlinear function) to simulate experimental data (Fig 4c and S1 Fig). The simulation results suggest that the dynamic properties of potassium ion channels faithfully follow an exponential decay function. Therefore, we chose the exponential function for our model and all other simulations. Moreover, we examined several new model network configurations to test inverse correlations between t_1/e_ and [K^+^]_o_. First, we assessed gap junctions between astrocytes in the same neuron-astrocyte module (S2A Fig). In the simulation, we reduced the gap junction weight to 6.5 in order to match previous experimental parameters. The simulation results of our novel model (S2C Fig) are indeed quite similar to our original model with only one gap junction in a two module network (S2B Fig). Therefore, either there are more gap junctions with a weak weight between glial cells or fewer gap junctions with a strong gap junction weight, and the network model reproduced established experimental results. Choosing 1:4 as the ratio of neurons to glial cells might be closer to actual physiology [33]. Second, we scaled up the network to a relatively large network with more neurons and glia, as well as increased gap junctions (same gap junction weight as the original model in S3A and S3B Fig). The simulation results show that scaled up large networks produced similar experimental decay curves as the two module small network (S3D and S3E Fig). Hence, these results suggest that the gap junction form and basic network configuration are robustly represented in this model.

It is well established that astrocyte dysfunction causes hyper-excitation and the generation or spread of seizure activity; dysfunctional astrocytes should be considered promising targets for new therapies. This study aimed to provide an in-depth understanding of Kir4.1 channels and astrocytic gap junctions in regulating the extracellular K^+^ microenvironment during epileptic seizures in order to facilitate construction of more accurate and dynamic models of neuron-astrocyte networks to improve recognition, forecasting and control of epileptic seizures.

## Materials and methods

### Neuron model

It is assumed that neurons have different local architectures. Each excitatory neuron model has *I*_*Na*_, *I*_*K*_, leak current *I*_*L*_ [43], after-hyperpolarization current *I*_*AHP*_. The H-H-type dynamic equations for the two neurons are as follows:

$$C\frac{dV_{N}}{dt}=-I_{Na}-I_{K}-I_{L}-I_{AHP}+I_{ext}$$ (1)

where, C is the membrane capacitance. The voltage-gated currents *I*_*Na*_ and *I*_*K*_, the leak current *I*_*L*_, and the after-hyperpolarization current *I*_*AHP*_ in Eq (1) are:

$$\begin{array}{l} I_{Na}=-g_{Na}m^{3}h(V_{N}-V_{Na})\\ I_{K}=-g_{K}n^{4}(V_{N}-V_{K})\\ I_{AHP}=-(\frac{g_{AHP}{\left[Ca\right]}_{i}}{1+{\left[Ca\right]}_{i}})(V_{N}-V_{K})\\ I_{L}=-g_{NaL}(V_{N}-V_{Na})--g_{KL}(V_{N}-V_{K})-g_{Cl}(V_{N}-V_{Cl}) \end{array}$$ (2)

Where *g*_*Na*_ and *g*_*K*_ denote the conductances corresponding to the sodium and potassium currents, respectively. *g*_*AHP*_ is the conductance corresponding to the after-hyperpolarization current. *g*_*NaL*_ and *g*_*KL*_ are the conductances corresponding to the sodium leak current and potassium leak current, respectively. *V*_*Na*_, *V*_*K*_ and *V*_*Cl*_ denote the Na^+^, K^+^ and Cl^-^ channel reversal potentials, respectively. *n*, *m* and *h* are gating variables for sodium and potassium currents. *g*_*Ca*_ and *V*_*Ca*_ are the conductance and the reversal potential of calcium, respectively. [Ca]_i_ corresponds to the intracellular calcium concentration, and the dynamics equation is:

$$\frac{d{\left[Ca\right]}_{i}}{dt}=-0.002g_{Ca}(V_{N}-V_{Ca})/\{1+\text{exp}(-(V_{N}+25)/2.5)\}-{\left[Ca\right]}_{i}/80$$ (3)

The equations for the gating variables in Eq (2) are:

$$\frac{dq}{dt}=\varphi [\alpha_{q}(V_{N})(1-q)-\beta_{q}(V_{N})q],\;\;\;\;\;\;q=m,n,h$$ (4)

$$\begin{array}{l} \alpha_{m}=0.1(V_{N}+30)/[1-\text{exp}(-0.1(V_{N}+30))]\\ \beta_{m}=4\;\text{exp}[-(V_{N}+55)/18\text{]}\\ \alpha_{n}=0.01(V_{N}+34)/[1-\text{exp}(-0.1(V_{N}+34))]\\ \beta_{n}=0.125\;\text{exp}(-(V_{N}+44)/80)\\ \alpha_{h}=0.07\;\text{exp}(-(V_{N}+44)/20)\\ \beta_{h}=1/[1+\text{exp}(-0.1(V_{N}+14))] \end{array}$$

The reversal potentials of Na^+^ and K^+^ and Cl^-^ are given by the Nernst equation:

$$\begin{array}{c} V_{Na}=26.64\;\text{ln}(\frac{{\left[Na\right]}_{o}}{{\left[Na\right]}_{Ni}})\\ V_{Cl}=26.64\;\text{ln}(\frac{{\left[Cl\right]}_{Ni}}{{\left[Cl\right]}_{o}})\\ V_{K}=26.64\;\text{ln}(\frac{{\left[K\right]}_{o}}{{\left[K\right]}_{Ni}}) \end{array}$$ (5)

where [*Na*]_*Ni*_ and [*Na*]_*o*_ denote the sodium ion concentrations in the intra-neuronal and extraneuronal spaces, respectively, The reversal potential of the Cl^-^ current is equal to a fixed value, that is, *V*_*Cl*_ = −81.93 mV. [*K*^+^]_*Ni*_ and [*K*^+^]_*o*_ denote the potassium concentration in the intra-neuronal and extraneuronal spaces, respectively.

### Balance of ions fluxes

The [*K*]_*o*_ value is continuously updated by the K^+^ currents across the neuronal membrane, Na^+^/K^+^-ATP pumps of neuron, K^+^ spatial diffusion [6,23,48], Na^+^/K^+^-ATP pumps and Kir4.1 channels of four astrocytes. The electrical current through cell membrane can cause ionic concentration of the inside and outside cell changing. A electrical current *I* across a membrane is equal to ion flow per unit of time. Thus, the K^+^ concentration dynamics for the neurons and astrocytes and the extra-neuronal space are described as follows:

$$\begin{array}{c} \frac{d{\left[K^{+}\right]}_{o}}{dt}=J_{IK}-2J_{pump,N}-2J_{pump,A1}-2J_{pump,A2}-2J_{pump,A3}-2J_{pump,A4}\\ +J_{Kir,1}+J_{Kir,2}+J_{Kir,3}+J_{Kir,4}-J_{diff} \end{array}$$ (6)

$$\frac{d{\left[K^{+}\right]}_{A,i}}{dt}=(-J_{kir,i}+2J_{pumpA,i}\text{)}v_{rate2}$$ (7)

$$\frac{d{\left[K^{+}\right]}_{N}}{dt}=(-J_{IK}+2J_{pump,N})v_{rate1}$$ (8)

Similar to the K^+^ dynamics, The [*Na*]_*o*_ value is continuously updated by the Na^+^ currents across the neuronal membrane, Na^+^/K^+^-ATPase pumps of neuron [6,23], Na^+^/K^+^-ATP pumps of four astrocytes. However, the main differences are that the pump exchanges two K^+^ for three Na^+^ ions, leading to the coefficient 3 in front of the pump term. In addition to sodium concentrations was added two constant leak terms *J*_*NaLA*_ and *J*_*NaLN*_, Thus, the Na^+^ concentration dynamics for neurons and astrocytes and the extraneuronal space are modeled as follows:

$$\begin{array}{c} \frac{d{\left[Na^{+}\right]}_{o,i}}{dt}=J_{Na,N}+3J_{pump,N}+3J_{pump,A1}+3J_{pump,A2}+3J_{pump,A3}\\ +3J_{pump,A4}+J_{NaL,N}+3J_{Na\text{L},A} \end{array}$$ (9)

$$\frac{d{\left[Na^{+}\right]}_{A,i}}{dt}=(-3J_{pump,Ai}-J_{Na\text{L},Ai})v_{rate2}$$ (10)

$$\frac{d{\left[Na\right]}_{N}}{dt}=(-J_{Na,N}-3J_{pump,N}-J_{NaL,N}\text{)}v_{rate1}$$ (11)

Also, different terms in the expressions (6–11) are described as follows:

$$\begin{array}{l} J_{pump,N}=\rho (\frac{1}{1.0+\text{exp}(25.0-{\left[Na\right]}_{Ni})/3.0})\times (\frac{1}{1+\text{exp}(8-{\left[K\right]}_{o})})\\ J_{diff}=\varepsilon ({\left[K\right]}_{o}-k_{bath})\\ J_{pump,Ai}=(\frac{1}{3})\rho (\frac{1}{1.0+\text{exp}(25.0-{\left[Na\right]}_{Ai})/3.0})\times (\frac{1}{1+\text{exp}(8-{\left[K\right]}_{o})}) \end{array}$$ (12)

where, *ρ* is the pump strength of Na^+^/K^+^-ATP, [*Na*]_*Ni*_ and [*Na*]_*Ai*_ are the sodium concentration for neuron and astrocyte, respectively, *ε* is the spatial diffusion coefficient of K^+^, and *K*_*bath*_ is the K^+^ concentration in the largest nearby reservoir.

### Model of membrane potential and Kir4.1 channels of astrocytes

The equation of the astrocyte membrane potential *V*_*A*_ is:

CAdVAdt=−Ikir−IAL (13)

Where *C*_*A*_ is the astrocytic capacitance, the leak current *I*_*IA*_=g_*IA*_(*V*_*A*_-*V*_*IA*_). The inward rectifier Kir4.1 channels current *I*_*kir*_ in astrocyte depends on the membrane potential and the extracellular potassium ion concentration, the expression can be described as [31,32]:

$$\begin{array}{c} I_{kirA}=g_{kir}\sqrt{{\left[K^{+}\right]}_{o}}(V_{A}-V_{kirA})\\ V_{kirA}=E_{kir}\text{log}({\left[K^{+}\right]}_{o}/{\left[K^{+}\right]}_{A}) \end{array}$$ (14)

Where *g*_*kir*_ and *E*_*kir*_ are the conductance and the Nernst constant for the astrocyte Kir 4.1 channels, respectively.

### Astrocytic coupling

The K^+^ dynamics in two astrocytes coupled by gap junction is described as follows:

$$\frac{d{\left[K^{+}\right]}_{Ai}}{dt}=-J_{kir,i}-2J_{pumpA,i}-J_{gap,k,ij}$$ (15)

The electrical current through the cell membrane can cause the ionic concentrations inside and outside of the cell to change. An electrical current *I* across a membrane is equal to the ion flow per unit of time. We convert the electrical current I into the ionic flux *J*, which is computed from *J* = *I*/(C*γ), where *C* is the astrocytic membrane capacitance. Here, *γ* is the scaling factor that relates the ion flux to the membrane potential.

where *J*_*gap*,*k*,*ij*_ is the potassium ions flow model mediated by astrocytic gap junction:

$$\begin{array}{ll} J_{gap,k}= & \{\begin{array}{cc} F/(\theta (\Delta_{i,j}K)\cdot e^{\left(-(\theta (\Delta_{i,j}K\text{-}K_{Thre})\text{/}\tau )\right)}\text{)} & \Delta_{i,j}K>K_{Thre}\\ 0 & \Delta_{i,j}K<K_{Thre} \end{array} \end{array}$$ (16)

where *F* is the strength of gap junction between astrocytes. *θ*Δ_*ij*_*K* is the K^+^ concentration gradient for two adjacent astrocytes. The threshold function *θ*(*x*) = *x* if *x* > *0*, and 0 otherwise. The astrocytic gap junction mediated K^+^ buffer allows astrocytic network to respond to changes of the extracellular K^+^ concentration.

The values of the parameters used in the model are listed in Table 1.

In simulating the numerical results of gap junction dysfunction that induces extracellular K^+^ buffering delay, we assumed that neuron 1 (N1) and neuron 2 (N2) share the same potassium bath concentration (in other words, neuron 1 (N1) and neuron 2 (N2) have the same value of *K*_*bath*_ in Eq (12)). The 4th-order Runge-Kutta method was used for numerical simulation with a time step of *h* = *0*.*01 ms*. Additionally, parameter values used in the numerical simulation are shown in Table 1, assuming no special emphasis.

## Supporting information

S1 Fig A. The relationship between extracellular K^+^ concentration and the decay factor t_1/e_ for experimental data (gray open circles, fitted curve is shown in the gray solid line) and the model data with an exponential function (red open circles, fitted curve is shown in the red line). B. The relationship between extracellular K^+^ concentration and decay factor t_1/e_ for experimental data (gray open circles, fitted curve is shown in the gray solid line) and the model data with a linear diffusion function (blue open circles, fitted curve is shown in the blue line). C. The relationship between extracellular K^+^ concentration and decay factor t_1/e_ for experimental data (gray open circles, fitted curve is shown in the gray solid line) and the model data with a threshold-nonlinear diffusion function (green open circles, fitted curve is shown in the green line). D. The statistical difference between the experimental and model data fitted curves for the three model functions are shown in the bar graph, with red for exponential function, blue for linear diffusion function, and green for threshold-nonlinear function.
(TIFF)

S2 Fig A. A conceptual diagram of the astrocytic-neural network model where gap junctions exist between astrocytes surrounding neurons. B. The relationship between extracellular K^+^ concentration and decay factor t_1/e_ for experimental data (gray open circles, fitted curve is shown in the gray solid line) and the model data with an exponential function (red open circles, fitted curve is shown in the red line). C. The relationship between extracellular K^+^ concentration and decay factor t_1/e_ for experimental data (gray open circles, fitted curve is shown in the gray solid line) and the model data with an exponential diffusion function in astrocytes around neurons (blue open circles, fitted curve is shown in the blue line). The other parameters used are the same as in Fig 1.
(TIFF)

S3 Fig A. A conceptual diagram of a 2*3 astrocytic-neural modulus network model with exponential function diffusion. B. A conceptual diagram of a 4*3 astrocytic-neural modulus network model with exponential function diffusion. C. The relationship between extracellular K^+^ concentration and decay factor t_1/e_ for experimental data (gray open circles, fitted curve is shown in the gray solid line) and the model data with an exponential function (red open circles, fitted curve is shown in the red line) in a two module network. D. The relationship between extracellular K^+^ concentration and decay factor t_1/e_ for experimental data (gray open circles, fitted curve is shown in the gray solid line) and the model data with an exponential diffusion function (blue open circles, fitted curve is shown in the blue line) in a 2*3 modulus network. E. The relationship between extracellular K^+^ concentration and decay factor t1/e for experimental data (gray open circles, fitted curve is shown in the gray solid line) and the model data with an exponential diffusion function (green open circles, fitted curve is shown in the green line) in a 4*3 modulus network.
(TIFF)

S1 Text Model details for simulating the inverse correlation relationship between t_1/e_ and [K^+^]_o_ with different diffusion function and network configurations. (DOCX)

## References

[1] Prinz AA. Understanding epilepsy through network modeling. Proc Natl Acad Sci U S A. 2008;105: 5953–5954. doi: 10.1073/pnas.0802299105 18413596 10.1073/pnas.0802299105 PMC2329695

[2] Traynelis SF, Dingledine R. Potassium-induced spontaneous electrographic seizures in the rat hippocampal slice. J Neurophysiol. 1988;59: 259–276. doi: 10.1152/jn.1988.59.1.259 3343603 10.1152/jn.1988.59.1.259

[3] Bikson M, Hahn PJ, Fox JE, Jefferys JGR. Depolarization block of neurons during maintenance of electrographic seizures. J Neurophysiol. 2003;90: 2402–2408. doi: 10.1152/jn.00467.2003 12801897 10.1152/jn.00467.2003

[4] Bazhenov M, Timofeev I, Steriade M, Sejnowski TJ. Potassium model for slow (2–3 Hz) in vivo neocortical paroxysmal oscillations. J Neurophysiol. 2004;92: 1116–1132. doi: 10.1152/jn.00529.2003 15056684 10.1152/jn.00529.2003 PMC2925854

[5] Fröhlich F, Timofeev I, Sejnowski TJ. 26–Extracellular Potassium Dynamics and Epileptogenesis. Computational Neuroscience in Epilepsy. 2008 pp. 419–439.

[6] Ullah G, Schiff SJ. Assimilating seizure dynamics. PLoS Comput Biol. 2010;6: e1000776 doi: 10.1371/journal.pcbi.1000776 20463875 10.1371/journal.pcbi.1000776 PMC2865517

[7] Ohno Y, Tokudome K, Kunisawa N. Role of astroglial Kir4.1 channels in the pathogenesis and treatment of epilepsy. 《Therapeutic Targets Neurol Dis. 2014; 1–10.

[8] Kucheryavykh YV, Kucheryavykh LY, Nichols CG, Maldonado HM, Baksi K, Reichenbach A, et al Downregulation of Kir4.1 inward rectifying potassium channel subunits by RNAi impairs potassium transfer and glutamate uptake by cultured cortical astrocytes. Glia. 2007;55: 274–281. doi: 10.1002/glia.20455 17091490 10.1002/glia.20455

[9] Largent-Milnes TM, Hegarty DM, Aicher SA, Andresen MC. Physiological temperatures drive glutamate release onto trigeminal superficial dorsal horn neurons. J Neurophysiol. 2014;111: 2222–2231. doi: 10.1152/jn.00912.2013 24598529 10.1152/jn.00912.2013 PMC4097869

[10] Du M, Li J, Wang R, Wu Y. The influence of potassium concentration on epileptic seizures in a coupled neuronal model in the hippocampus. Cogn Neurodyn. 2016;10: 405–414. doi: 10.1007/s11571-016-9390-4 27668019 10.1007/s11571-016-9390-4 PMC5018011

[11] Kofuji P, Newman EA. Potassium buffering in the central nervous system. Neuroscience. 2004;129: 1045–1056. 15561419 10.1016/j.neuroscience.2004.06.008 PMC2322935

[12] Somjen GG. Ion regulation in the brain: implications for pathophysiology. Neurosci Rev J Bringing Neurobiol Neurol Psychiatry. 2002;8: 254–267. doi: 10.1177/1073858402008003011 12061505 10.1177/1073858402008003011

[13] Butt AM, Kalsi A. Inwardly rectifying potassium channels (Kir) in central nervous system glia: a special role for Kir4.1 in glial functions. J Cell Mol Med. 2006;10: 33–44. doi: 10.1111/j.1582-4934.2006.tb00289.x 16563220 10.1111/j.1582-4934.2006.tb00289.x PMC3933100

[14] Heuser K, Eid T, Lauritzen F, Thoren AE, Vindedal GF, Taubøll E, et al Loss of perivascular Kir4.1 potassium channels in the sclerotic hippocampus of patients with mesial temporal lobe epilepsy. J Neuropathol Exp Neurol. 2012;71: 814–825. doi: 10.1097/NEN.0b013e318267b5af 22878665 10.1097/NEN.0b013e318267b5af PMC3470834

[15] Larsen BR, Assentoft M, Cotrina ML, Hua SZ, Nedergaard M, Kaila K, et al Contributions of the Na^+^/K^+^-ATPase, NKCC1, and Kir4.1 to hippocampal K^+^ clearance and volume responses. Glia. 2014;62: 608–622. doi: 10.1002/glia.22629 24482245 10.1002/glia.22629 PMC4302754

[16] Steinhäuser C, Seifert G, Bedner P. Astrocyte dysfunction in temporal lobe epilepsy: K+ channels and gap junction coupling. Glia. 2012;60: 1192–1202. doi: 10.1002/glia.22313 22328245 10.1002/glia.22313

[17] Wallraff A, Köhling R, Heinemann U, Theis M, Willecke K, Steinhäuser C. The impact of astrocytic gap junctional coupling on potassium buffering in the hippocampus. J Neurosci Off J Soc Neurosci. 2006;26: 5438–5447. doi: 10.1523/JNEUROSCI.0037-06.2006 16707796 10.1523/JNEUROSCI.0037-06.2006 PMC6675300

[18] Pannasch U, Derangeon M, Chever O, Rouach N. Astroglial gap junctions shape neuronal network activity. Commun Integr Biol. 2012;5: 248–254. doi: 10.4161/cib.19410 22896785 10.4161/cib.19410 PMC3419107

[19] Seifert G, Steinhäuser C. Neuron-astrocyte signaling and epilepsy. Exp Neurol. 2013;244: 4–10. doi: 10.1016/j.expneurol.2011.08.024 21925173 10.1016/j.expneurol.2011.08.024

[20] Bedner P, Steinhäuser C. Altered Kir and gap junction channels in temporal lobe epilepsy. Neurochem Int. 2013;63: 682–687. doi: 10.1016/j.neuint.2013.01.011 23357483 10.1016/j.neuint.2013.01.011

[21] Xu L, Zeng L-H, Wong M. Impaired astrocytic gap junction coupling and potassium buffering in a mouse model of tuberous sclerosis complex. Neurobiol Dis. 2009;34: 291–299. doi: 10.1016/j.nbd.2009.01.010 19385061 10.1016/j.nbd.2009.01.010 PMC2764295

[22] Kovács R, Heinemann U, Steinhäuser C. Mechanisms underlying blood-brain barrier dysfunction in brain pathology and epileptogenesis: role of astroglia. Epilepsia. 2012;53 Suppl 6: 53–59. doi: 10.1111/j.1528-1167.2012.03703.x 23134496 10.1111/j.1528-1167.2012.03703.x

[23] Cressman JR, Ullah G, Ziburkus J, Schiff SJ, Barreto E. The influence of sodium and potassium dynamics on excitability, seizures, and the stability of persistent states: I. Single neuron dynamics. J Comput Neurosci. 2009;26: 159–170. doi: 10.1007/s10827-008-0132-4 19169801 10.1007/s10827-008-0132-4 PMC2704057

[24] David Y, Cacheaux LP, Ivens S, Lapilover E, Heinemann U, Kaufer D, et al Astrocytic dysfunction in epileptogenesis: consequence of altered potassium and glutamate homeostasis? J Neurosci Off J Soc Neurosci. 2009;29: 10588–10599. doi: 10.1523/JNEUROSCI.2323-09.2009 19710312 10.1523/JNEUROSCI.2323-09.2009 PMC2875068

[25] Wei Y, Ullah G, Schiff SJ. Unification of neuronal spikes, seizures, and spreading depression. J Neurosci Off J Soc Neurosci. 2014;34: 11733–11743. doi: 10.1523/JNEUROSCI.0516-14.2014 25164668 10.1523/JNEUROSCI.0516-14.2014 PMC4145176

[26] Wei Y, Ullah G, Ingram J, Schiff SJ. Oxygen and seizure dynamics: II. Computational modeling. J Neurophysiol. 2014;112: 213–223. doi: 10.1152/jn.00541.2013 24671540 10.1152/jn.00541.2013 PMC4064403

[27] Kager H, Wadman WJ, Somjen GG. Simulated seizures and spreading depression in a neuron model incorporating interstitial space and ion concentrations. J Neurophysiol. 2000;84: 495–512. doi: 10.1152/jn.2000.84.1.495 10899222 10.1152/jn.2000.84.1.495

[28] Øyehaug L, Østby I, Lloyd CM, Omholt SW, Einevoll GT. Dependence of spontaneous neuronal firing and depolarisation block on astroglial membrane transport mechanisms. J Comput Neurosci. 2012;32: 147–165. doi: 10.1007/s10827-011-0345-9 21667153 10.1007/s10827-011-0345-9

[29] Østby I, Øyehaug L, Einevoll GT, Nagelhus EA, Plahte E, Zeuthen T, et al Astrocytic mechanisms explaining neural-activity-induced shrinkage of extraneuronal space. PLoS Comput Biol. 2009;5: e1000272 doi: 10.1371/journal.pcbi.1000272 19165313 10.1371/journal.pcbi.1000272 PMC2613522

[30] Hübel N, Ullah G. Anions Govern Cell Volume: A Case Study of Relative Astrocytic and Neuronal Swelling in Spreading Depolarization. PloS One. 2016;11: e0147060 doi: 10.1371/journal.pone.0147060 26974767 10.1371/journal.pone.0147060 PMC4790933

[31] Sibille J, Dao Duc K, Holcman D, Rouach N. The neuroglial potassium cycle during neurotransmission: role of Kir4.1 channels. PLoS Comput Biol. 2015;11: e1004137 doi: 10.1371/journal.pcbi.1004137 25826753 10.1371/journal.pcbi.1004137 PMC4380507

[32] Witthoft A, Filosa JA, Karniadakis GE. Potassium buffering in the neurovascular unit: models and sensitivity analysis. Biophys J. 2013;105: 2046–2054. doi: 10.1016/j.bpj.2013.09.012 24209849 10.1016/j.bpj.2013.09.012 PMC3824545

[33] Azevedo FAC, Carvalho LRB, Grinberg LT, Farfel JM, Ferretti REL, Leite REP, et al Equal numbers of neuronal and nonneuronal cells make the human brain an isometrically scaled-up primate brain. J Comp Neurol. 2009;513: 532–541. doi: 10.1002/cne.21974 19226510 10.1002/cne.21974

[34] Di Garbo A. Dynamics of a minimal neural model consisting of an astrocyte, a neuron, and an interneuron. J Biol Phys. 2009;35: 361–382. doi: 10.1007/s10867-009-9143-2 19669428 10.1007/s10867-009-9143-2 PMC2750740

[35] Chever O, Djukic B, McCarthy KD, Amzica F. Implication of Kir4.1 channel in excess potassium clearance: an in vivo study on anesthetized glial-conditional Kir4.1 knock-out mice. J Neurosci Off J Soc Neurosci. 2010;30: 15769–15777. doi: 10.1523/JNEUROSCI.2078-10.2010 21106816 10.1523/JNEUROSCI.2078-10.2010 PMC6633770

[36] Ballanyi K, Grafe P, ten Bruggencate G. Ion activities and potassium uptake mechanisms of glial cells in guinea-pig olfactory cortex slices. J Physiol. 1987;382: 159–174. 2442359 10.1113/jphysiol.1987.sp016361 PMC1183018

[37] Heinemann U, Lux HD. Undershoots following stimulus-induced rises of extracellular potassium concentration in cerebral cortex of cat. Brain Res. 1975;93: 63–76. 1139318 10.1016/0006-8993(75)90286-3

[38] Elisevich K, Rempel SA, Smith BJ, Edvardsen K. Hippocampal connexin 43 expression in human complex partial seizure disorder. Exp Neurol. 1997;145: 154–164. 9184118 10.1006/exnr.1997.6467

[39] Collignon F, Wetjen NM, Cohen-Gadol AA, Cascino GD, Parisi J, Meyer FB, et al Altered expression of connexin subtypes in mesial temporal lobe epilepsy in humans. J Neurosurg. 2006;105: 77–87. doi: 10.3171/jns.2006.105.1.77 16874892 10.3171/jns.2006.105.1.77

[40] Fonseca CG, Green CR, Nicholson LFB. Upregulation in astrocytic connexin 43 gap junction levels may exacerbate generalized seizures in mesial temporal lobe epilepsy. Brain Res. 2002;929: 105–116. 11852037 10.1016/s0006-8993(01)03289-9

[41] Ingram JM, Zhang C, Xu J, Schiff SJ. FRET excited ratiometric oxygen sensing in living tissue. J Neurosci Methods. 2013;214: 45–51. doi: 10.1016/j.jneumeth.2013.01.002 23333398 10.1016/j.jneumeth.2013.01.002 PMC3664065

[42] Ziburkus J, Cressman JR, Barreto E, Schiff SJ. Interneuron and pyramidal cell interplay during in vitro seizure-like events. J Neurophysiol. 2006;95: 3948–3954. doi: 10.1152/jn.01378.2005 16554499 10.1152/jn.01378.2005 PMC1469233

[43] Ullah G, Cressman JR, Barreto E, Schiff SJ. The influence of sodium and potassium dynamics on excitability, seizures, and the stability of persistent states. II. Network and glial dynamics. J Comput Neurosci. 2009;26: 171–183. doi: 10.1007/s10827-008-0130-6 19083088 10.1007/s10827-008-0130-6 PMC2951284

[44] Neusch C, Papadopoulos N, Müller M, Maletzki I, Winter SM, Hirrlinger J, et al Lack of the Kir4.1 channel subunit abolishes K+ buffering properties of astrocytes in the ventral respiratory group: impact on extracellular K+ regulation. J Neurophysiol. 2006;95: 1843–1852. doi: 10.1152/jn.00996.2005 16306174 10.1152/jn.00996.2005

[45] Harada Y, Nagao Y, Mukai T, Shimizu S. Expressional Analysis of Inwardly Rectifying Kir4.1 Channels in Groggy Rats, a Rat Model of Absence Seizures. Arch Neurosci. 2014;

[46] Bay V, Butt AM. Relationship between glial potassium regulation and axon excitability: a role for glial Kir4.1 channels. Glia. 2012;60: 651–660. doi: 10.1002/glia.22299 22290828 10.1002/glia.22299

[47] Pannasch U, Vargová L, Reingruber J, Ezan P, Holcman D, Giaume C, et al Astroglial networks scale synaptic activity and plasticity. Proc Natl Acad Sci U S A. 2011;108: 8467–8472. doi: 10.1073/pnas.1016650108 21536893 10.1073/pnas.1016650108 PMC3100942

[48] Wendling F, Bartolomei F, Mina F, Huneau C, Benquet P. Interictal spikes, fast ripples and seizures in partial epilepsies—combining multi-level computational models with experimental data. Eur J Neurosci. 2012;36: 2164–2177. doi: 10.1111/j.1460-9568.2012.08039.x 22805062 10.1111/j.1460-9568.2012.08039.x

