# Supplementary figures and images for: Astrocytic Kir4.1 channels and gap junctions account for spontaneous epileptic seizure
Source: PLoS Comput Biol. 2018 Mar 28;14(3):e1005877. doi: 10.1371/journal.pcbi.1005877 (PMC5891073; doi:10.1371/journal.pcbi.1005877)

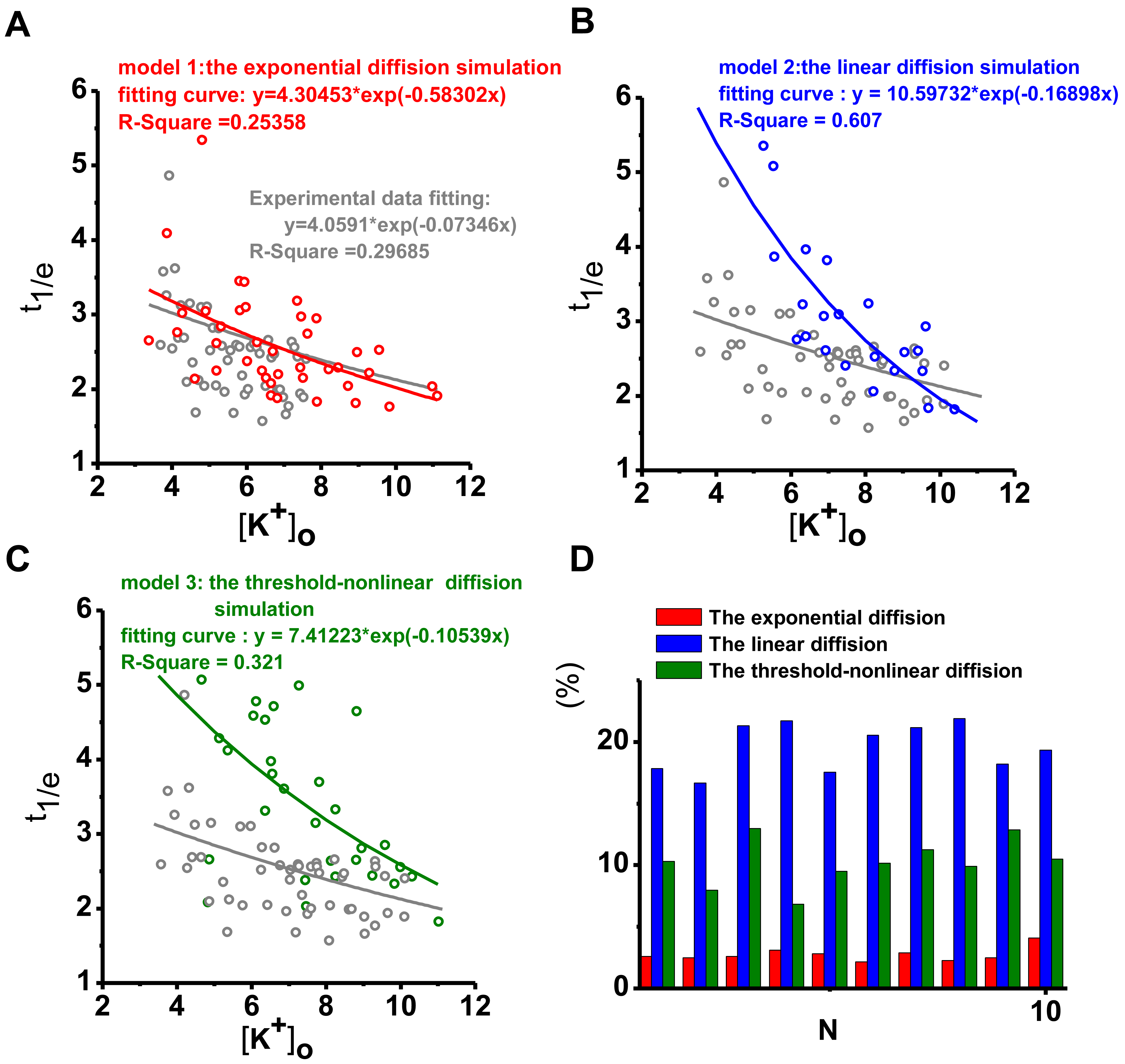

Supplement: S1 Fig — A. The relationship between extracellular K+ concentration and the decay factor t1/e for experimental data (gray open circles, fitted curve is shown in the gray solid line) and the model data with an exponential function (red open circles, fitted curve is shown in the red line). B. The relationship between extracellular K+ concentration and decay factor t1/e for experimental data (gray open circles, fitted curve is shown in the gray solid line) and the model data with a linear diffusion function (blue open circles, fitted curve is shown in the blue line). C. The relationship between extracellular K+ concentration and decay factor t1/e for experimental data (gray open circles, fitted curve is shown in the gray solid line) and the model data with a threshold-nonlinear diffusion function (green open circles, fitted curve is shown in the green line). D. The statistical difference between the experimental and model data fitted curves for the three model functions are shown in the bar graph, with red for exponential function, blue for linear diffusion function, and green for threshold-nonlinear function. (TIFF) [file pcbi.1005877.s001.tiff]

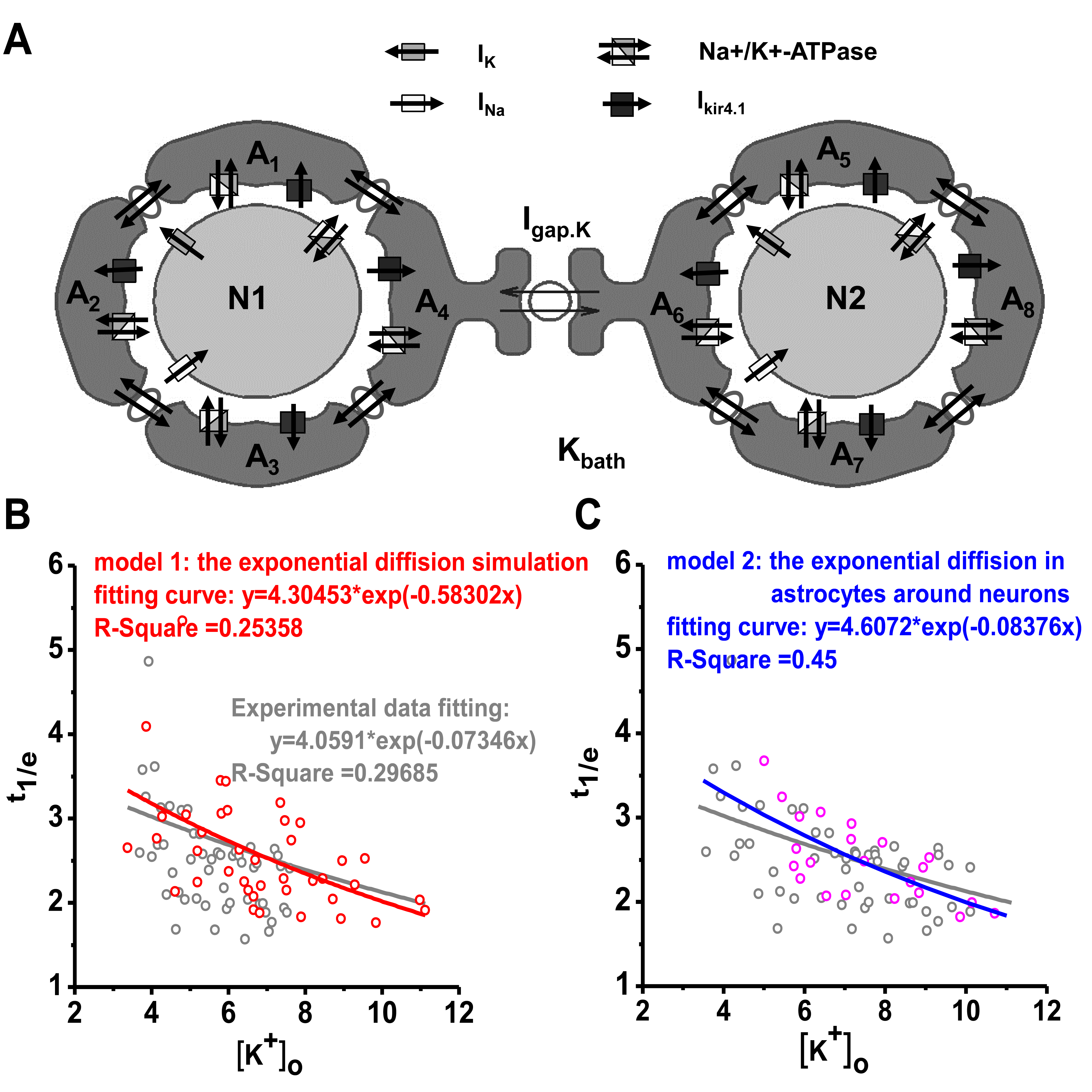

Supplement: S2 Fig — A. A conceptual diagram of the astrocytic-neural network model where gap junctions exist between astrocytes surrounding neurons. B. The relationship between extracellular K+ concentration and decay factor t1/e for experimental data (gray open circles, fitted curve is shown in the gray solid line) and the model data with an exponential function (red open circles, fitted curve is shown in the red line). C. The relationship between extracellular K+ concentration and decay factor t1/e for experimental data (gray open circles, fitted curve is shown in the gray solid line) and the model data with an exponential diffusion function in astrocytes around neurons (blue open circles, fitted curve is shown in the blue line). The other parameters used are the same as in Fig 1. (TIFF) [file pcbi.1005877.s002.tiff]

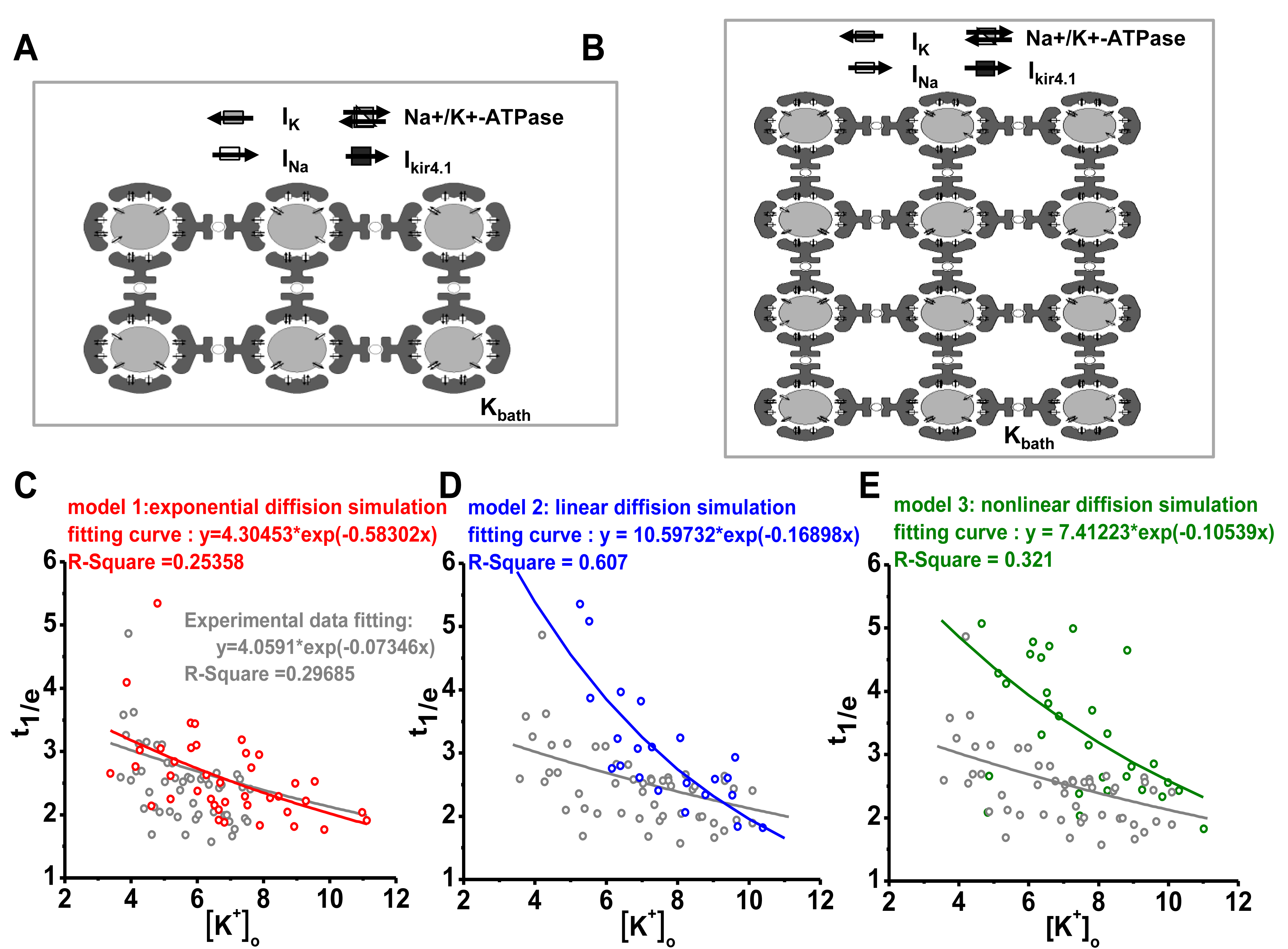

Supplement: S3 Fig — A. A conceptual diagram of a 2*3 astrocytic-neural modulus network model with exponential function diffusion. B. A conceptual diagram of a 4*3 astrocytic-neural modulus network model with exponential function diffusion. C. The relationship between extracellular K+ concentration and decay factor t1/e for experimental data (gray open circles, fitted curve is shown in the gray solid line) and the model data with an exponential function (red open circles, fitted curve is shown in the red line) in a two module network. D. The relationship between extracellular K+ concentration and decay factor t1/e for experimental data (gray open circles, fitted curve is shown in the gray solid line) and the model data with an exponential diffusion function (blue open circles, fitted curve is shown in the blue line) in a 2*3 modulus network. E. The relationship between extracellular K+ concentration and decay factor t1/e for experimental data (gray open circles, fitted curve is shown in the gray solid line) and the model data with an exponential diffusion function (green open circles, fitted curve is shown in the green line) in a 4*3 modulus network. (TIFF) [file pcbi.1005877.s003.tiff]
