# Supplementary material for: Astrocytic Kir4.1 channels and gap junctions account for spontaneous epileptic seizure
Source: PLoS Comput Biol. 2018 Mar 28;14(3):e1005877. doi: 10.1371/journal.pcbi.1005877 (PMC5891073; doi:10.1371/journal.pcbi.1005877)
Supplement: S1 Text — (DOCX) [file pcbi.1005877.s004.docx]

**SUPPLEMENTARY: Material and Methods**

Here, we describe the detailed models for simulating the inverse correlation relationship between t_1/e_ and [K^+^]_o_ with different diffusion function and network configurations.

**SI Text, Section A – The different diffusion function**

In this section, we provide three gap junction diffusion function simulation. Such as, the exponential function (See Eq. 16 in original paper), and the linear function (Eq. S1), and the threshold-nonlinear function (Eq.S2).

$$J_{ij}=F([K^{+}]_{Ai}-[K^{+}]_{Aj})\text{ (S1)}$$

Where, *i* ,*j* are astrocytes 4 and astrocyte 6 in Figure 3. *F* is the strength of gap junction between astrocytes. *K_thr_* = 0.05*mM* and *K_Scale_* = 1*mM*. The H-H-type dynamic equations for the two neurons are also already given in the original paper. In simulation, the Kir4.1 channel conductance is set to 45.0 *pS* and the other parameters used are same as Figure 1. We set the weight *F* are 11 and 10.8 to better match the experimental fitting results as using linear function (Eq. S1) and threshold-nonlinear function (Eq. S2), respectively.

Moreover, the fitting error (in Figure S1D) is adopted by the mean absolute error ($\frac{1}{\text{n}}\sum_{1}^{n} (\frac{q_{i}-p_{i}}{q_{i}})\times100\%$). {*p_i_*} and {*q_i_*} are points of the simulated fitting results and experimental result (see Fig. 5B from [Wallraff A, 2006]).), respectively. *n* is the fitting times as the K^+^ concentration *K_bath_*, the spatial diffusion coefficient *ε* and the Na^+^/K^+^ -ATP pump strength *ρ* are set to different values.

**SI Text, Section A.1 - Adding gap junctions between astrocytes in the same neuron-astrocyte module**

In this section, we add gap junctions between astrocytes in the same neuron-astrocyte module but same exponential function gap junction as original model, see Figure S2A. The H-H-type dynamic equations for the two neurons are also already given in the original paper. K^+^ concentration dynamics in each astrocyte surrounding neurons are

Where, *i* and *j* presents astrocytes 1-3 and astrocytes 5,7,8 in Figure S2A. $J_{diff,ij}\text{ }$and are diffusion ion fluxs between nearest astrocytes surrounding neurons, which is described by equation (16) in original paper. Moreover, K^+^ concentration dynamics in astrocytes 4 and astrocytes 6 in Figure S2A are:

In simulation, the Kir4.1 channel conductance is set to 45.0 *pS* and the other parameters used are same as Figure 1. We reduce the gap junction weight to 6.5 to match the experimental results. The simulation results of the new model (see Figure S2C) are indeed quite similar to our original model with only one gap junction in two module network (see Figure S2B).

**SI Text, Section A.2 - The network with more gap junctions but same gap junction weight as original model**

Here, we scaled up the network to be a relatively large network with more gap junctions but same gap junction weight as original model (see Figure S3A, and 3B). The H-H-type dynamic equations for the two neurons are also already given in the original paper. K^+^ concentration dynamics in each astrocyte surrounding neurons are:

$$\frac{d[K^{+}]_{A,i,j1}}{dt}=(-J_{kir,i,j1}+2J_{pumpA,i,j1}\text{)}v_{rate2\text{ }}\text{ }-\text{ }J_{diff,i,j1\text{;}i-1\text{,}j,3}\text{ (S6)}$$

$$\frac{d[K^{+}]_{A,i,j,2}}{dt}=(-J_{kir,i,j,2}+2J_{pumpA,i,j,2}\text{)}v_{rate2\text{ }}\text{ }-\text{ }J_{diff,i,j,2\text{;}i\text{,}j-1,4}\text{ (S7)}$$

$$\frac{d[K^{+}]_{A,i,j\text{，}3}}{dt}=(-J_{kir,i,j\text{，}3}+2J_{pumpA,i,j\text{，}3}\text{)}v_{rate2\text{ }}\text{ }-\text{ }J_{diff,i,j\text{，}3\text{;}i+1\text{,}j,1}\text{ (S8)}$$

$$\frac{d[K^{+}]_{A,i,j\text{,}4}}{dt}=(-J_{kir,i,j\text{，}4}+2J_{pumpA,i,j\text{，}4}\text{)}v_{rate2\text{ }}\text{ }-\text{ }J_{diff,i,j\text{，4;}i\text{,}j+1,2}\text{ (S9)}$$

Where, *J_kir_* and *J_pumpA_* are same as original model in original paper. The exponential functions *J_diff_* are gap junction diffusion ion flux between astrocytes in network. and gap junctions weight are same as original model. In simulation, the Kir4.1 channel conductance is set to 45.0 pS and the other parameters used are same as Figure 1. The simulation results show that scaled up large network could reproduce the similar experimental decay curve as the two-module small network (see Figure S3D, and 3E).

**SUPPLEMENTARY: Figures**

Fig.S1. A, the relations of extracellular K^+^ concentration and decay factor *t_1/e_* for experimental data (gray open circle, fitting curve is shown in gray solid line) and the model data with exponential function (red open circle, fitting curve is shown in red line). B, the relations of extracellular K^+^ concentration and decay factor *t_1/e_* for experimental data (gray open circle, fitting curve is shown in gray solid line) and the model data with linear diffusion function (blue open circle, fitting curve is shown in blue line). C, the relations of extracellular K^+^ concentration and decay factor *t_1/e_* for experimental data (gray open circle, fitting curve is shown in gray solid line) and the model data with threshold-nonlinear diffusion function (green open circle, fitting curve is shown in green line). (D) the statistical difference between the experimental and model data fitting curves for the three model functions are shown in bar plot with color red for exponential function, blue for linear diffusion function, and green for threshold-nonlinear function, respectively.

####

#### Fig.S2. A, A conceptual diagram of the astrocytic-neural network model with gap junctions existing between astrocytes surrounding neurons. B, the relations of extracellular K^+^ concentration and decay factor *t_1/e_* for experimental data (gray open circle, fitting curve is shown in gray solid line) and the model data with exponential function (red open circle, fitting curve is shown in red line). C, the relations of extracellular K^+^ concentration and decay factor *t_1/e_* for experimental data (gray open circle, fitting curve is shown in gray solid line) and the model data with the exponential diffusion function in astrocytes around neurons (blue open circle, fitting curve is shown in blue line).

####

#### Fig.S3. A, A conceptual diagram of 2*3 astrocytic-neural modulus network model with the exponential diffusion function. B, A conceptual diagram of 4*3 astrocytic-neural modulus network model with the exponential function diffusion. C. the relations of extracellular K^+^ concentration and decay factor *t_1/e_* for experimental data (gray open circle, fitting curve is shown in gray solid line) and the model data with exponential function (red open circle, fitting curve is shown in red line) in two modulus network. D, the relations of extracellular K^+^ concentration and decay factor *t_1/e_* for experimental data (gray open circle, fitting curve is shown in gray solid line) and the model data with exponential diffusion function (blue open circle, fitting curve is shown in blue line) in 2*3 modulus network. E, the relations of extracellular K^+^ concentration and decay factor *t_1/e_* for experimental data (gray open circle, fitting curve is shown in gray solid line) and the model data with exponential diffusion function (green open circle, fitting curve is shown in green line) in 4*3 modulus network.
